# *QuickStats*: Rate* of Deaths Attributed to Unintentional Injury from Fire or Flames,† by Sex and Urban-Rural Status§ — National Vital Statistics System, United States, 2020

**DOI:** 10.15585/mmwr.mm7114a5

**Published:** 2022-04-08

**Figure:**
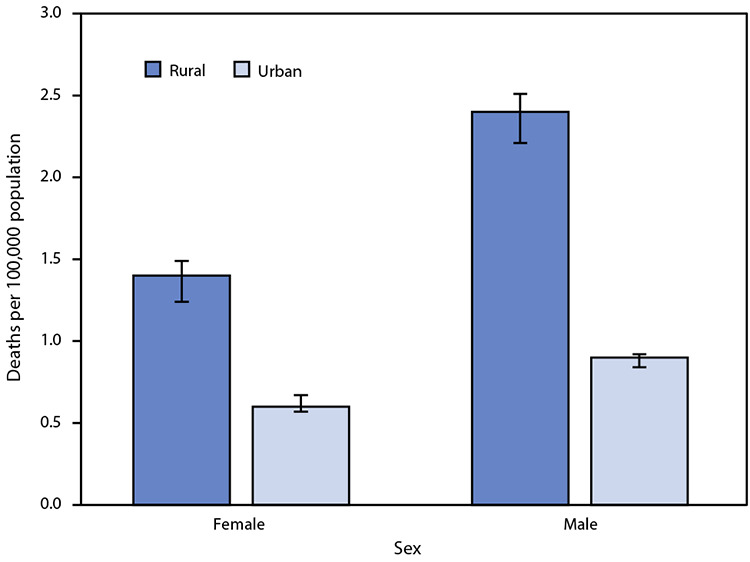
In 2020, the death rate attributed to unintentional injury from fire or flames was higher in rural areas than in urban areas for females and males. The rate for females was 1.4 per 100,000 in rural areas and 0.6 in urban areas. The rate for males was 2.4 per 100,000 in rural areas and 0.9 in urban areas. Males had higher death rates than females in both rural and urban areas.

**Source:** National Center for Health Statistics, National Vital Statistics System, Mortality Data, 2020. https://www.cdc.gov/nchs/deaths.htm

